# Supplementary material for: In vivo super-resolution RESOLFT microscopy of Drosophila melanogaster
Source: eLife. 2016 Jun 29;5:e15567. doi: 10.7554/eLife.15567 (PMC4927295; doi:10.7554/eLife.15567)

**Supplementary table 1. List of colormaps used.**

The numbers indicate the photon counts that correspond to the lowest and highest value of the respective colormap. Blue color indicates saturation.


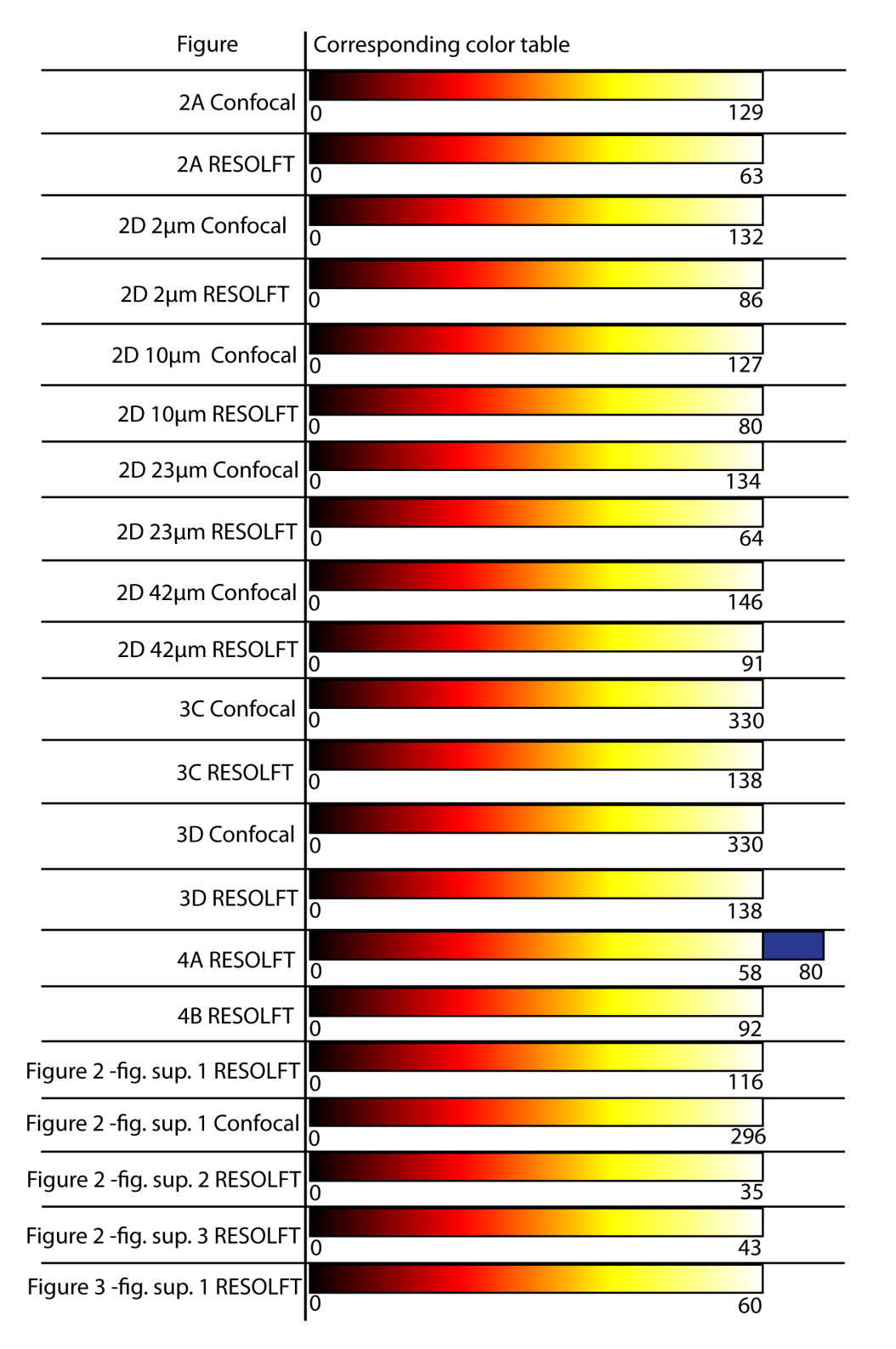

Supplement: Supplementary file 1. — The numbers indicate the photon counts that correspond to the lowest and highest value of the respective color map. Blue color indicates saturation. DOI: http://dx.doi.org/10.7554/eLife.15567.016 [file elife-15567-supp1.docx]
