# Supplementary material for: In vivo super-resolution RESOLFT microscopy of Drosophila melanogaster
Source: eLife. 2016 Jun 29;5:e15567. doi: 10.7554/eLife.15567 (PMC4927295; doi:10.7554/eLife.15567)
Supplement: Supplementary file 2. — Light powers were measured in front of the objective’s back focal plane. DOI: http://dx.doi.org/10.7554/eLife.15567.017 [file elife-15567-supp2.docx]

**Supplementary table 2. Imaging parameters used for RESOLFT microscopy.**

Light powers were measured in front of the objective back focal plane.

| **RESOLFT-image** | **405 nm on switching** | | **488 nm off switching beam** | | **488 nm readout** | | **Pixel size xy [nm]** | **Scanning step size z [nm]** | **Line accumulation** |
| --- | --- | --- | --- | --- | --- | --- | --- | --- | --- |
|  | Power [µW] | Illumination time [µs] | Power [µW] | Illumination time [µs] | Power [µW] | Illumination time [µs] |  |  |  |
| 2 A | 4.1 | 20 | 28 | 430 | 11.9 | 30 | 25 |  | 2 |
| 2D | 1.5 | 17 | 26 | 380 | 14.4 | 10 | 20 |  | 2 |
| 3 C | 11 | 20 | 32 | 520 | 16.9 | 30 | 25 |  | 3 |
| 4 A | 2.5 | 20 | 39 | 300 | 9.4 | 40 | 30 |  | / |
| 4 B | 1.35 | 15 | 40 | 180 | 16.9 | 20 | 35 |  | / |
| Movie 1 | 2.9 | 10 | 15.4 | 450 | 6.8 | 40 | 25 | 250 | 2 |
| Movie 2 | 2.9 | 20 | 36 | 490 | 10.2 | 40 | 35 | 60 | / |
| Movie 3 | 2.5 | 20 | 39 | 300 | 9.4 | 40 | 30 |  | / |
| Movie 4 | 1.35 | 15 | 40 | 180 | 16.9 | 20 | 35 |  | / |
| Figure 2-figure supplement 1 | 11 | 20 | 20.5 | 500 | 11.9 | 30 | 25 |  | 3 |
| Figure 2-figure supplement 2 | 2.9 | 10 | 15.4 | 450 | 6.8 | 40 | 25 | 250 | 2 |
| Figure 2-figure supplement 3 | 2.9 | 20 | 36 | 490 | 10.2 | 40 | 35 | 60 | / |
| Figure 3-figure supplement 1 | 11 | 20 | 34 | 520 | 14.4 | 10 | 20 |  | 3 |
